# Differential Modulation of Heat-Inducible Genes Across Diverse Genotypes and Molecular Cloning of a sHSP From Pearl Millet [*Pennisetum glaucum* (L.) R. Br.]

**DOI:** 10.3389/fpls.2021.659893

**Published:** 2021-07-16

**Authors:** S. Mukesh Sankar, C. Tara Satyavathi, Sharmistha Barthakur, Sumer Pal Singh, C. Bharadwaj, S. L. Soumya

**Affiliations:** ^1^Division of Genetics, Indian Council of Agricultural Research-Indian Agricultural Research Institute, New Delhi, India; ^2^Indian Council of Agricultural Research-All India Coordinated Research Project on Pearl Millet (AICPMIP), Jodhpur, India; ^3^Functional Genomics, Indian Council of Agricultural Research-National Institute for Plant Biotechnology (NIPB), New Delhi, India

**Keywords:** Thermotolerance, Heat Shock Proteins, alpha-crystalline domain, Pearl Millet, Cloning, qRT-PCR, RNA-seq

## Abstract

The survival, biomass, and grain yield of most of the crops are negatively influenced by several environmental stresses. The present study was carried out by using transcript expression profiling for functionally clarifying the role of genes belonging to a small heat shock protein (sHSP) family in pearl millet under high-temperature stress. Transcript expression profiling of two high-temperature-responsive marker genes, *Pgcp70* and *PgHSF*, along with physio-biochemical traits was considered to screen out the best contrasting genotypes among the eight different pearl millet inbred lines in the seedling stage. Transcript expression pattern suggested the existence of differential response among different genotypes upon heat stress in the form of accumulation of heat shock-responsive gene transcripts. Genotypes, such as WGI 126, TT-1, TT-6, and MS 841B, responded positively toward high-temperature stress for the transcript accumulation of both *Pgcp70* and *PgHSF* and also indicated a better growth under heat stress. PPMI-69 showed the least responsiveness to transcript induction; moreover, it supports the membrane stability index (MSI) data for scoring thermotolerance, thereby suggesting the efficacy of transcript expression profiling as a molecular-based screening technique for the identification of thermotolerant genes and genotypes at particular crop growth stages. The contrasting genotypes, such as PPMI-69 (thermosusceptible) and WGI-126 and TT-1 (thermotolerant), are further utilized for the characterization of thermotolerance behavior of sHSP by cloning a *PgHSP16.97* from the thermotolerant cv. WGI-126. In addition, the investigation was extended for the identification and characterization of 28 different HSP20 genes through a genome-wide search in the pearl millet genome and an understanding of their expression pattern using the RNA-sequencing (RNA-Seq) data set. The outcome of the present study indicated that transcript profiling can be a very useful technique for high-throughput screening of heat-tolerant genotypes in the seedling stage. Also, the identified *PgHSP20s* genes can provide further insights into the molecular regulation of pearl millet stress tolerance, thereby bridging them together to fight against the unpredicted nature of abiotic stress.

## Introduction

Pearl millet [*Pennisetum glaucum* (L.) R. Br.] is an annual warm season, C_4_ cereal crop that is cultivated extensively in the Indian subcontinent and African semiarid regions. It covers an area of 29 million ha and forms the staple food and fodder for 90 million resource-poor inhabitants (Sharma et al., 2021). With a total area of 7.41 million ha, India is the largest producer of pearl millet in the world, having an annual production of 10.3 million tons in 2020–2021 (Indiastat, 2020). Pearl millet is mostly grown as a *Kharif* crop with the least input during the hottest periods by the onset of monsoon. A high temperature of more than 42°C accompanied by moisture stress during the seedling stage of the crop, particularly at germination and seedling establishment, affects the plant status and thereby its productivity. A rise in air and soil temperature of the semiarid regions of India and sub-Saharan Africa due to global climate change continues to be a severe constraint in the growth and development of the crop; thereby causing a huge loss in terms of quantity and quality (Ullah et al., 2018). Such a situation demands critical attention not only for the development of stress-tolerant genotypes but also for the identification and characterization of genes responsible for thermotolerance.

Plants being sessile can dramatically alter their gene expressions in response to various stress signals through a set of morphological, physiological, and molecular changes that can harm the stability of plant growth and productivity (Castelán-Muñoz et al., 2019). The activation and regulation of stress-related genes play a significant role in the acquisition of thermotolerance among plants. In response to heat stress, pearl millet produces an array of proteins that help in alleviating the effects of stress. One such major protein family is heat shock proteins (HSPs). Both stress signal transduction and gene activation are aided by HSPs/chaperones (Nievola et al., 2017). Heat stress-responsive signal transduction pathways and defense mechanisms involve heat shock factors (HSFs) and HSPs in sensing reactive oxygen species (ROS) produced under heat stress and in preventing protein misfolding (Sajid et al., 2018). For the first time, Howarth. C. J. (1991) reported that specific HSP transcripts aid in the diagnosis of plant stress and play a role in the acquisition of thermotolerance in pearl millet. Based on their approximate molecular weights, five major families of HSPs, namely HSP100, HSP90, HSP70, HSP60, and the HSP20 or small HSP (sHSP), are recognized (Waters and Vierling, 2020). HSP20s are the diverse groups of proteins that are preserved in both eukaryotes and prokaryotes with molecular weights in the range of 11–35 kDa, the expression of which is limited in the absence of environmental stress but upregulated to over 200-fold upon the induction of heat stress (Wahid et al., 2007). By protecting the native proteins from irreversible aggregation and oxidative inactivation, it plays a decisive role in the defense of an organism during physiological stress (Ma et al., 2021). One of the research findings further showed that sHSPs, along with HSP70, play a key role in quality control, possibly contributing to the maintenance of cell membrane integrity, especially under stress (Savic et al., 2012). Hence, the maintenance of cell membrane fluidity under high-temperature stress is due to a higher expression of these two major genes, its expression pattern across genotypes can be used for screening seedling thermotolerance.

HSP20 gene families have been reported in *Arabidopsis*, rice, tomato, potato, watermelon, apple, grapes, and some other plant species (Scharf et al., 2001; Ouyang et al., 2009; Sarkar et al., 2009; Yu et al., 2016; Zhao et al., 2018; He et al., 2019; Ji et al., 2019; Yao et al., 2020). Furthermore, HSP20 genes are organized into multigenic families, with defined classification and biological functions (Waters et al., 1996; Scharf et al., 2001; Siddique et al., 2008; Sarkar et al., 2009; Zhang et al., 2016). Although the available pearl millet whole-genome sequence is a vital asset for an in-depth understanding of various gene families distributed among the genomes (Varshney et al., 2017), little knowledge on the integrated HSP20 family at the whole genomic level in pearl millet exists. The research findings will assist the researcher to truly assess the functional role of the pearl millet HSP20 gene family and its role in stress adaptation. These genes can pose a great opportunity in the development of a thermotolerant cultivar in pearl millet and other cereals.

In conventional breeding for heat tolerance, the identification of reliable and effective screening methods to facilitate the identification of heat-tolerant plants and the genes responsible for thermotolerance represents a major challenge. The transcript expression method based on semi-quantitative reverse transcription- (RT-) PCR offers a rapid and cost-effective way to screen out large germplasm lines or advance breeding lines that were inferior under specific thermal regimes to more accurate methods such as quantitative real-time PCR (qRT-PCR) or RNA-sequencing (RNA-Seq). Recently, various researchers employed semi-quantitative RT-PCR for the successful screening of germplasm and for fishing out the candidate genes related to drought and heat stresses (Bharadwaj et al., 2019; Choi et al., 2019; Galvez et al., 2020).

In this context, the current study aimed (1) to screen the most contrasting genotypes against heat stress using the physiological and biochemical parameters, (2) to confirm the results of the abovementioned screening techniques with the semi-quantitative RT-PCR expression profile of key heat-responsive marker genes, such as *Pgcp70* and *PgHSF*, (3) to identify, clone, and characterize a *PgHSP20* gene from the most thermotolerant genotype, and (4) to distinguish various members of the pearl millet *HSP20* gene family using a bioinformatics method and to characterize the integration of sequence features, chromosome location, phylogenetic relationship, and expression patterns. This study unravels the effectiveness of reverse transcription PCR- (RT-PCR) based screening methods for the identification and utilization of the thermotolerant genes from the superior heat-tolerant genotypes to bridge the supra-optimal temperature tolerance with high productivity in pearl millet.

## Materials and Methods

### Plant Materials and Growth Conditions

A total of eight elite pearl millet inbred lines (six thermotolerant and two thermosusceptible), based on our earlier studies (Mukesh Sankar et al., 2014; James et al., 2015), were collected from the pearl millet breeding unit of the Indian Agricultural Research Institute (IARI), New Delhi, India. The pedigree, salient characteristics, and breeding station from where it has been developed are presented in Table 1. The experiments were done in a completely randomized block design with three replications. Seeds were surface sterilized and grown in a pot containing autoclaved Soilrite™ and were kept with a 16-h photoperiod at 25°C in a culture room as control until subjecting them to heat stress treatment in a growth chamber (Supplementary Figure 1).

**Table 1:** Details of genotypes used for transcript expression studies for heat tolerance in pearl millet.

**Sl. No**	**Genotype**	**Codes**	**Pedigree, salient characteristics, and breeding use**	**Seed source^**a**^**	**Mean MSI (%)^**b**^**
1	MS 411 B	G1	Extra early male sterile line developed from 263 B through selection.	IARI	56.38
2	MS 841 B	G2	DM-resistant selection from residual variability in 5141B; seed parent of Pusa 23, Pusa 322, and Pusa 605	IARI-ICRISAT	71.21
3	D-23	G3	DM-resistant derivative of K-560-230; Male parent of Pusa 23	IARI	65.84
4	PPMI-69	G4	Derivative of PPMI 43; Male parent of Pusa 605	IARI	52.53
5	PPMI-301	G5	Derivative of a cross between four elite restorers having bold ear head; Male parent of Pusa 322	IARI	67.29
6	TT-1	G6	Selection from line no. 868 suited for arid regions of Jodhpur	CAZRI	67.99
7	TT-6	G7	Selection from line no. 873 suited for arid regions of Jodhpur	CAZRI	70.65
8	WGI-126	G8	Pearly white, bold grained inbred with sturdy stem and broad leaf	IARI	67.65

a *CAZRI, Central Arid Zone Research Institute, Jodhpur (India); ICRISAT, International Crop Research Institute for SemiArid Tropics, Hyderabad (India); IARI, Indian Agricultural Research Institute, New Delhi (India)*.

b *Mean MSI value*.

*Source: Mukesh Sankar et al. (2014) (MSI > 65% were thermotolerant)*.

### Screening of Genotypes Using Physiological and Biochemical Parameters

Preliminary analysis of the already known physiological and biochemical marker traits for seedling thermotolerance was undertaken to determine the most thermotolerant and thermosusceptible ones among the eight genotypes. Hence, 10-day-old seedlings were subjected to heat stress at 42°C for 6 h.

The membrane stability index (MSI) of both control and stressed seedlings was assessed by using the modified method given by Sairam and Tyagi (2004) with the formula:

$$\begin{array}{r} \text{MSI }\left(\text{\%}\right)=\left[1-\left({\text{C}}_{1}/{\text{C}}_{2}\right)\right]\text{×}100 \end{array}$$

Relative water content (RWC) of both control and stressed seedlings was estimated by applying the formula:

$$\begin{array}{r} \text{RWC }\left(\text{\%}\right)=\left[\left(\text{FW}-\text{DW}\right)/\left(\text{TW}-\text{DW}\right)\right]\text{×}100 \end{array}$$

as described by Yang et al. (2011).

Leaf absorbance was measured with a chlorophyll meter (SPAD-502, Minolta, Tokyo, Japan) as a non-destructive tool for estimating leaf chlorophyll. The lipid peroxidation level for all the samples was estimated through the thiobarbituric acid-reactive substance (TBARS) assay proposed by Heath and Packer (1968).

### Heat Stress Treatment, RNA Isolation, and Transcript Expression Profiling

Heat stress was imposed on 7- and 10-day-old seedlings in a controlled growth chamber at 42°C for 2 and 6 h, respectively. The aerial portion of seedlings was harvested after heat treatment given in a growth chamber. Simultaneously, another set of plant samples kept as control were also collected under controlled conditions. To preserve the stage-specific transcript, all collected tissue samples were wrapped in an aluminum foil, labeled, and flash-frozen by using liquid nitrogen before being stored at −80°C for RNA isolation. Total RNA was isolated from the tissues using the TRIzol reagent (Invitrogen, Carlsbad, CA, USA). The isolated RNAs were checked in 1.2% agarose gel. About 1 μg of the samples were then treated with DNAse (Promega Corporation, Madison, WI, USA) and subjected to NanoDrop spectrophotometer quantification (ND-1000, Thermo Fisher Scientific, Waltham, MA, USA). DNAse-treated RNA was reverse transcribed by using the Thermo, Verso^TM^cDNA-kit (Thermo Fisher Scientific Inc., Waltham, MA, USA). For each reaction, 50 ng of complementary DNA (cDNA) was used as a template, accompanied by the amplification with gene-specific primers.

The transcript expression patterns of two well-known pearl millet genes, namely HSP70 and HSF, were compared to identify the best thermotolerant and thermosusceptible ones among the selected pearl millet genotypes in response to heat stress. The Pearson correlation coefficient was also estimated to conclude the relationship among the studied physiological and biochemical traits with the gene expression level of the candidate genes. Coding sequences of these two representative pearl millet genes, i.e., *Pgcp70* (Acc. no. EF495353.1) and *Pghsf* (Acc. no. EU492460.1), were downloaded from NCBI (http://www.ncbi.nlm.nih.gov). Specific primers were designed to amplify the selected messenger RNA (mRNA) sequence of the abovementioned genes for the semiquantitative RT-PCR expression analysis while maintaining stringency and specificity. The details of the primers along with their melting temperatures (Tm) are presented in Table 2. RT-PCR reactions were then performed by using the PCR master mix (Promega Corporation, Madison, WI, USA) as per the instruction of the manufacturer in a volume of 50-μl reaction mixture. A total of 30 cycles of PCR with 4 min of an initial denaturation at 94°C, 94°C for 45 s, 48–60°C (Tm optimized for the individual gene) for 45 s, 72°C for 1-min amplification with a final extension at 72°C for 10 min were performed. The RT-PCR products were loaded on 1.2% agarose gel, and the stained DNA products were photographed by using Alfa Imager gel documentation system HP (Protein Simple, San Jose, CA, USA). In all expression studies, the housekeeping gene actin was used as a reference gene (internal constitutive control) to show equal loading of cDNA and to ensure its integrity under various degrees of heat stress. The transcript levels of each target gene were averaged over three reactions. The gene expression data were normalized by deducting the mean expression level from the reference gene. The relative fold change in the expression of treatments (T) was compared with those from the regular growth stage (C) and done by the expression value of the susceptible genotype control as a calibrator for the respective genotype using Alfa Imager Software tools by keeping the density of bands in the control as unity.

**Table 2:** Details of primers used for transcript expression profiling and full-length cloning by reverse transcription PCR (RT-PCR).

**Gene name**	**Strand**	**Primer Sequence (5^**′**^->3^**′**^)**	**Expected product size**	**Tm**
*Pgcp70*	F	5' ACAGGGAAGAAGCAGGACATGACA 3'	184 bp	50.0°C
	R	5' AGCTCCTTGAGTTGCTTCTCGGTT 3'		
*PgHSF*	F	5' ATATCTTCGCCTCCCTCAGGGTGATA 3'	145 bp	48.0°C
	R	5' GTATGAAGGCAACACACCACGCAA 3'		
Pg actin	F	5' AGCGAGTCTTCATAGGGCGATTGT 3'	200 bp	60.0°C
	R	5' TAGCTCTGGGTTCGAGTGGCATTT 3'		
*PgHSP16.97*	F	5' AGTTTCAGCAATGTCGCTGGT 3'	560 bp	53.0°C
	R	5' ACAAGCACGACTCGTAGCATC 3'		

*Where F, indicates the forward primer and R, indicates the reverse primer*.

### Isolation and Cloning of a Full-Length *PgHSP16.97*

The full-length ORF of *PgHSP16.97* was amplified from the pearl millet inbred genotype, WGI 126, by using the primers designed based on the sequence homology with other grasses sequences available in the public domain (Table 2). The sequence was cloned in a pGMT vector, confirmed by colony PCR and restriction digestion followed by Sanger sequencing (Xcelris Labs Ltd., Ahmedabad, India). The sequence homology was carried out by using NCBI (www.ncbi.nlm.nih.gov) tools and verified and deposited in GenBank. The conceptual translation of nucleotide sequence was undertaken by using the Expasy translate tool (http://web.expasy.org/translate/). Multiple sequence alignment were carried out by using the CLUSTALW software package (Thompson et al., 1994) with full-length HSP 17.0 protein sequences publicly available for *P. glaucum, Zea mays, Oryza sativa, Triticum aestivum, Sorghum bicolor*, and *Hordeum* spp.

### Expression Analysis of *PgHSP16.97* in Different Tissues at Different Developmental Stages

To investigate the expression pattern of HSP 16.97, the tissue samples from the whole seedling at 10 days after sowing (DAS), leaves, stems, and roots at 30 DAS, and flag leaf and developing panicle at 55 DAS were also collected from both control and heat-stressed plant at 42°C for 6 h. Semi-quantitative RT-PCR was carried out by using the same gene-specific primers for the cloning of the gene for an initial analysis. The GenScript Real-time PCR Primer design tool (https://www.genscript.com/ssl-bin/app/primer) was used to design the qRT-PCR primers (Table 3). The SYBR Green Master Mix (2X) was utilized according to the instruction of the manufacturer to study the gene expression (Thermo Fisher Scientific, Waltham, MA, USA). qRT-PCR analysis was performed in three technical replicates (obtained by pooling the tissues from the three biological samples of each treatment) and with the following thermal cycles: 1 cycle at 95°C for 30 s, followed by 40 cycles alternatively at 95°C for 5 s, and 58°C for 1 min in the StepOne Real-Time PCR system (Applied Biosystems, Waltham, MA, USA). Based on the comparative Ct method using the 2^−Δ*ΔCT*^ method, the relative expression levels of the PgHSP16.97 gene were estimated (Livak and Schmittgen, 2001). A constitutive *PgAct* gene-based primer was used as an endogenous control to normalize the number of transcripts accumulated for the PgHSP16.97 gene. The least expression value obtained from the control of the whole seedling in the sensitive line was considered as unity for relative quantification, and the relative fold changes in expression for the remaining lines and plant tissues were estimated.

**Table 3:** Details of gene-specific primers for qRT-PCR gene expression analysis.

**Gene name**	**Strand**	**Primer sequence (5^**′**^->3^**′**^)**	**Primer Tm (^**°**^C)**	**Primer length (bp)**	**Amplicon size (bp)**
*PgHSP16.97RT*	F	CAAGGCCGAGGAGAAGAAGC	57.8	20	118
	R	GCACGACTCGTAGCATCACA	57.6	20	
*Actin (PgAct)*	F	CTCAGTGGAGGATCTACTAT	59.4	20	108
	R	GGTGGTGCAATCACTTTAAC	63.2	20	

*Where F, indicates the forward primer and R, indicates the reverse primer*.

### Homology Modeling and Structure Analysis

A three-dimensional (3D) structure of PgHSP16.97 deduced by Modeller v9.11 (Sali and Blundell, 1993) was subjected to the backbone conformation evaluation by Ramachandran plot using Procheck (Laskowski et al., 1993). The final model and the template were subjected to superimposition for a structural comparison using the STRAP interface (http://www.bioinformatics.org/strap/).

### Genome-Wide *in silico* Database Mining for the Identification of HSP20 Proteins

To identify various other HSP20 genes present in *P. glaucum*, a total of 38,579 proteome sequences of *P. glaucum* were retrieved from the GIGA database (http://gigadb.org/dataset/100192). A Hidden Markow Model (HMM) profile of HSP20 (PF00011) downloaded from the Pfam v33.1 database (https://pfam.xfam.org/family/PF00011) was queried against the phytozome v.12.1 database of (https://phytozome.jgi.doe.gov/pz/portal.html) *Arabidopsis*, rice, maize, sorghum, and foxtail millet. All the resulted sequences which have a hit with expectation (E) <1.0 were retrieved in the construction of reference sequences (Supplementary Table 1). The reference sequences were aligned with default parameters by using the MUSCLE, and an HMM profile was built based on the obtained consensus of the aligned sequences using HMMER tool v3.2. By using the newly generated HMM profile, the proteome database of *P. glaucum* was scanned for probable homologous sequences in *P. glaucum* by using the HMMER search. Pfam and SMART (http://smart.embl-heidelberg.de/) were used to further confirm the conserved HSP20 domain. Finally, 28 HSP20s were identified after removing the redundant sequence without the conserved alpha-crystalline domain (ACD) domain of HSP20 and with the molecular weight being outside the range of 11–35 kDa. With the help of the Protparam online tools (https://web.expasy.org/protparam/), the physiochemical properties of HSP20 proteins were predicted.

### Chromosomal Location and Phylogenetic Analysis of HSP20

Based on the ascending order of chromosomal position from one end of the chromosome to the other end, PgHSP20 was annotated as PgHSP01 to PgHSP28 and the physical position was constructed by using MapInspect v1.0 (https://mapinspect.software.informer.com/1.0/). The representative HSP20 protein sequences from *Arabidopsis*, rice, tomato, and apple together with the PgHSP20 genes predicted from pearl millet were selected for the construction of a phylogenetic tree. A multiple sequence alignment of HSP20 proteins was conducted by using MUSCLE. The protein sequences were imported to MEGA X to construct a phylogenetic tree by using a maximum likelihood (MLE) method. The parameters utilized are bootstrapped for 1,000-bootstrap replications; substitution model: Jones–Taylor–Thornton (JTT) model with rates among the sites-gamma distributed (G) and partial deletion for gap/missing data treatment.

### Digital Expression Analysis of the Identified HSP 20 Genes

To understand the expression pattern of the identified PgHSP20 genes, the digital expression profiling of 28 identified genes was performed by using the Illumina-based whole transcriptome pearl millet RNA-Seq data set available in the NCBI database as the Sequence Read Archive (SRA) database under the accession number SRP151237. The Custom R script based on “annHeatmap” function in R package-Heatplus was utilized to decipher the expression profile of genes under heat stress with the thermotolerant genotype MS 841B and thermo-susceptible genotype PPMI 69 exposed to heat stress for 30 min and 6 h, respectively, at 42°C (Maibam et al., 2020).

### Statistical Analysis

The data from the physiological and biochemical parameters were initially analyzed to understand the extent of variability of the traits among the genotypes and the treatments using the ANOVA. The Duncan multiple range test (DMRT) was used for the separation of means within treatments and genotypes at a level of significance, *p* ≤ 0.05. One-way ANOVA was carried out to analyze the relative expression levels for various treatments in each genotype. The differences within treatments and genotypes were estimated by using DMRT (*p* ≤ 0.05). To compare the relative expression levels of PgHSP16.97 in the lines within/between the treatments for different plant parts, a *t*-test was used. All statistical analyses and assessment of the level of significance were carried out using the R software (v.3.6.2).

## Results

### Effects of Heat Stress on Various Physiological and Biochemical Traits of Pearl Millet Genotypes

**Figure 1:**
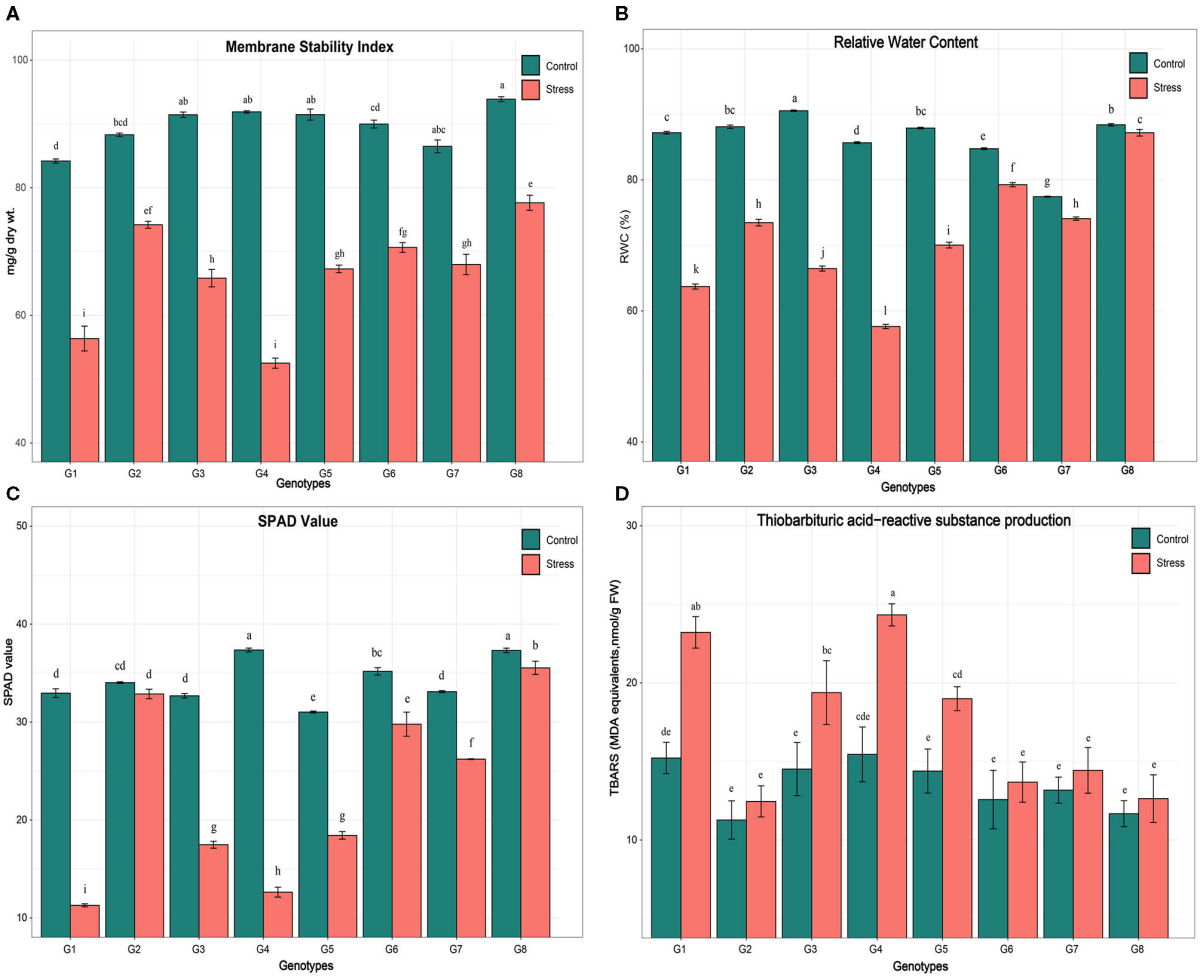
Effect of heat stress on **(A)** membrane stability index (MSI), **(B)** relative water content (RWC), **(C)** chlorophyll meter value (SPAD), **(D)** thiobarbaturtic acid-reactive substance level on eight pearl millet genotypes [MS 411B (G1), MS 841B (G2), D-23 (G3), PPMI-69 (G4), PPMI-301 (G5), TT-1 (G6), TT-6 (G7), and WGI-126 (G8)]. Mean value followed by the different alphabets for a parameter in different genotypes and treatments indicates a significant difference at *p* ≤ 0.05. Vertical bars show mean ± SE (https://figshare.com/s/dea2659a099b4158896c).

The ANOVA indicated a high and significant level of genetic variation (*p* < 0.001) among the genotypes for all physiological and biochemical traits under control and heat stress conditions (Table 4). The genotype PPMI-69 (G4) was found to be inferior in all heat stress-related parameters whereas WGI-126 (G8) and MS 841B (G2) showed more endurance to heat stress. Moreover, when heat stress was induced, traits such as MSI, RWC, SPAD values and TBARS levels varied widely. Under heat stress, MSI, RWC, and SPAD readings decreased on average for all genotypes, whereas the TBARS level increased. Furthermore, the treatment–genotype interaction was found to be highly significant (*p* < 0.001), indicating that the genotypes responded to heat stress differently. In pearl millet seedlings, heat stress caused statistically significant (*p* < 0.05) changes in the physiological and biochemical parameters (Figures 1A–D). Although there was a slight variation in the studied traits among the pearl millet genotypes under control conditions, they behaved very differently when heat stress was imposed. Upon the imposition of heat stress, MSI reduced significantly among all genotypes. However, the genotype G4 (PPMI-69) experienced a major fold reduction in MSI (42%) in comparison with the corresponding control, followed by G1 (MS 411B) and G3 (D-23). On the other hand, G8 (WGI-126) had the least reduction in MSI (17%), followed by MS 841B, upon heat stress.

**Table 4:** The mean square values of the treatments (T), genotypes (G), and the treatment–genotype interaction (T × G) along with their errors and significance indicated by the value of *p*.

**Source of variation**	**Df**	**MSI**		**RWC**		**SPAD**		**TBARS**	
		**MSS**	**Pr(>F)**	**MSS**	**Pr(>F)**	**MSS**	**Pr(>F)**	**MSS**	**Pr(>F)**
Treatment (T)	1	6122.45	0.000	2617.90	0.000	1498.96	0.000	178.59	0.000
Genotype (G)	7	282.00	0.000	143.17	0.000	157.11	0.000	59.58	0.000
Replication within Treatment (R)	4	5.55	0.443	0.38	0.254	0.62	0.417	4.30	0.544
T X G	7	97.97	0.000	160.40	0.000	118.35	0.000	15.81	0.021
Residuals	28	5.65		0.27		0.61		5.47	

A similar trend was observed with a significant decrease in RWC in all eight genotypes after imposing heat stress. Genotype G8 (WGI-126) had the least significant reduction in RWC followed by G6 (TT-1) and G2 (MS 841B). Heat stress on seedlings resulted in a significant reduction in chlorophyll content among the genotypes, as indicated by SPAD measurements. Differences were found to be significant (*p* ≤ 0.05) in genotypes G4 (PPMI-69) and G1 (MS 411B), under heat stress, with a reduction of 66 and 65%, respectively. On the other hand, G8 (WGI-126) and G6 (TT-1) had the least influence on SPAD reading under stress. A similar trend was followed by the production of TBARS upon oxidative stress due to heat stress in which G4 had the maximum oxidative damage and G8 with the least.

### Expression Pattern of Heat-Responsive Marker Genes Among Genotypes Under Different Regimes of Heat Stress

**Figure 2:**
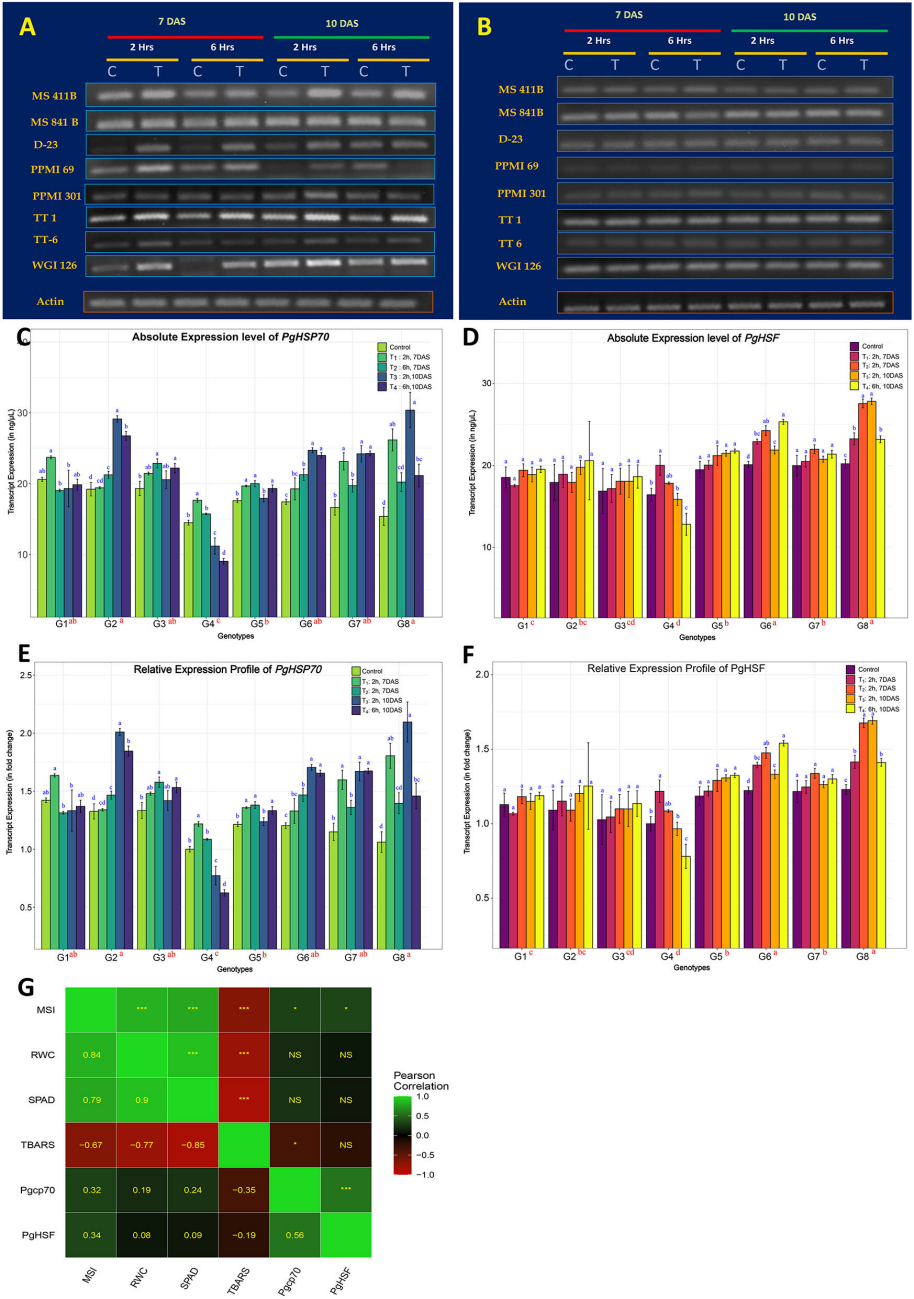
Heat-stress induced gene expression profile of eight pearl millet genotypes for two heat-responsive marker genes. **(A,B)** Reverse transcription PCR (RT-PCR) gel profiles of *Pgcp70* and *PgHSF* genes under regular plant growth (C) and those exposed to heat stress at 42°C (T) during different time periods of treatment indicated above in the seedling stage. The presented results are the representative of at least three independent biological replicates. **(C,D)** Comparative transcript expression profiling of *Pgcp70* and *PgHSF* for heat tolerance (mean ± SE) among eight pearl millet genotypes (considering absolute values) after normalization with that of actin gene (internal control). **(E,F)** The relative expression level of *Pgcp70* and *PgHSF* genes (mean ± SE) among eight genotypes under heat stress. For relative quantification, the least expression value from the whole seedling control in the sensitive genotype (PPMI-69) was considered as unity and the relative fold changes in expression were calculated for the remaining genotype and the treatments. **(G)** Association of marker gene expression values averaged for different heat treatments with physiological and biochemical parameters among different genotypes. Scale on the side of the figure indicates the magnitude and direction of correlations. Shades of green from darker to lighter indicate the strength of a positive correlation between pairs of traits. Shades of red from darker to lighter indicate the strength of a negative correlation between pairs of traits. Darker to black color indicates a very weak or no correlation between a pair of traits. Pearson's correlation coefficients (lower diagonal) and significance (upper diagonal) among different traits were measured in this study. ‘***' is *p* ≤ 0.001, ‘**' is *p* ≤ 0.01, ‘*' is *p* ≤ 0.05, and ‘NS' indicates non-significance associationship. Mean sharing the same alphabets for a parameter in different genotypes indicates that there is no significant difference at a significance level of *p* ≤ 0.05 (https://figshare.com/s/0c22f1e7599392059207).

The transcript expression patterns of the two well-studied pearl millet genes, HSP70 and HSF, in response to heat stress were compared to identify the best thermotolerant and thermosusceptible lines among the selected pearl millet genotypes in the transcript expression level. Responsiveness of genotype to stress in the seedling stage was also established. Hence, young seedlings of 7 days old and 10 days old were chosen, and heat stress of 42°C was imposed for 2 and 6 h, respectively. Comparisons were made within and between the genotypes with and without stress treatment. It was found that both gene transcripts were found to be differentially expressed, in all genotypes irrespective of the treatments and even under controlled conditions (Figures 2A–F). Pgcp70 got expressed under regular growth, the expression level of Pgcp70 was increased very significantly (*p* < 0.05) upon heat stress. The genotypes showed significant variability for transcript accumulation upon heat stress in which the level was upregulated by 68.9, 66.5, 57.6, 53.9, and 50.2%, respectively, in thermotolerant lines, such as WGI-126 (G8), MS 841B (G2), TT-6 (G7), TT-1 (G6), and D-23 (G3), whereas, in susceptible genotype PPMI-69 (G4), the gene was downregulated by 7.5% to susceptible control (Table 5). In MS 411B and PPMI-301, the percent change in expression was comparatively low. The genotype-wise high-temperature tolerance expression pattern of the stress-inducible gene Pgcp70 can be categorized as WGI-126 ≥ MS 841B > TT-1 ≥ TT-6 > D-23 > MS 411B > PPMI-301 > PPMI-69 based on the average relative expression level of transcript across the treatments. The expression profiling of Pgcp70 suggested that HSP 70 was highly induced at an early stage of heat exposure (for 2 h), the transcript level of which was slightly increased as heat stress progressed for a long duration of 6 h on 7-day-old seedlings. Even though we observed a constant induction of HSP 70 during heat stress on 10-day-old seedling, the result suggested that heat stress in the early phase (for 2 h) led to the upregulation of Pgcp70 transcript in all genotypes though its level is reduced with continuous exposure to heat stress (6 h) over a period. In addition, the transcript accumulation showed a slight increase from 7- to 10-day-old seedlings, indicating that the plant tends to increase its tolerance to high temperature with growth, development, and continued exposure.

**Table 5:** Comparison of the relative gene expression level of marker genes at seedling between different pearl millet genotypes under heat stress treatment (with level significance at *p* ≤ 0.05).

**S.N**	**Genotypes**	**Av.GE.Pgcp70**	**% change in expression**	Stat. Sig^*^	**Rank**	**Av.GE.PgHSF**	**% Change**	Stat. Sig^*^	**Rank**	**Cumulative rank**
1	MS 411B	1.413	41.3	bc	4	1.147	14.7	c	4	8
2	MS 841B	1.665	66.5	a	1	1.175	17.5	bc	3	4
3	D-23	1.502	50.2	abc	3	1.095	9.5	cd	5	8
4	PPMI-69	0.925	−7.5	d	6	1.013	1.3	d	6	12
5	PPMI-301	1.327	32.7	c	5	1.285	28.5	b	2	7
6	TT-1	1.539	53.9	ab	2	1.436	43.6	a	1	3
7	TT-6	1.576	57.6	ab	2	1.287	28.7	b	2	4
8	WGI-126	1.689	68.9	a	1	1.549	54.9	a	1	2

* *Indicates genotypes sharing the same letters are not significant; Av.GE means average gene expression level*.

In contrast to Pgcp70, only thermotolerant genotypes, such as G8 (WGI-126) and G6 (TT-1), and thermosusceptible genotypes, such as G4, were found to have a significant change in the PgHSF transcript expression, as indicated by the significance level (*p* < 0.05). The susceptible genotype, on the other hand, only showed a 1.3% increase in gene expression (Table 5). The transcript expression was found to increase upon extending the heat stress in most of the genotypes except PPMI-69. Genotypes can be categorized based on the *PgHSF* transcript abundance during the expression profiling as WGI-126 ≥ TT-1 > TT-6 ≥ PPMI-301 ≥ MS 841B ≥ MS 411B ≥ D-23 > PPMI-69. Based on the cumulative ranks, WGI-126 and TT-1 were considered as the best thermo-tolerant lines and PPMI-69 was considered as the most thermosusceptible line.

Moreover, the association of various physiological and biochemical parameters with the expression of heat stress marker genes holds a low-to-moderate significant relation (*r* > 0.3, *p* < 0.05) with MSI. TBARS had shown a significant low-to-moderate correlation with the gene expression Pgcp70. The physiological parameters, such as MSI, RWC, and SPAD values, have a high positive correlation with each other and show a strong negative correlation with the TBARS level upon heat stress. The expression of two markers upon heat stress was related by *r* = 0.56 (Figure 2G).

In general, considering the physiological and biochemical traits and gene expression of the heat-responsive marker gene, PPMI-69 (G4) was identified as the most thermosusceptible and WGI-126 (G8) as the most thermotolerant genotype among the eight genotypes undertaken in this study.

### Isolation and Cloning of Full-Length cDNA of PgHSP 17.0 From Pearl Millet

**Figure 3:**
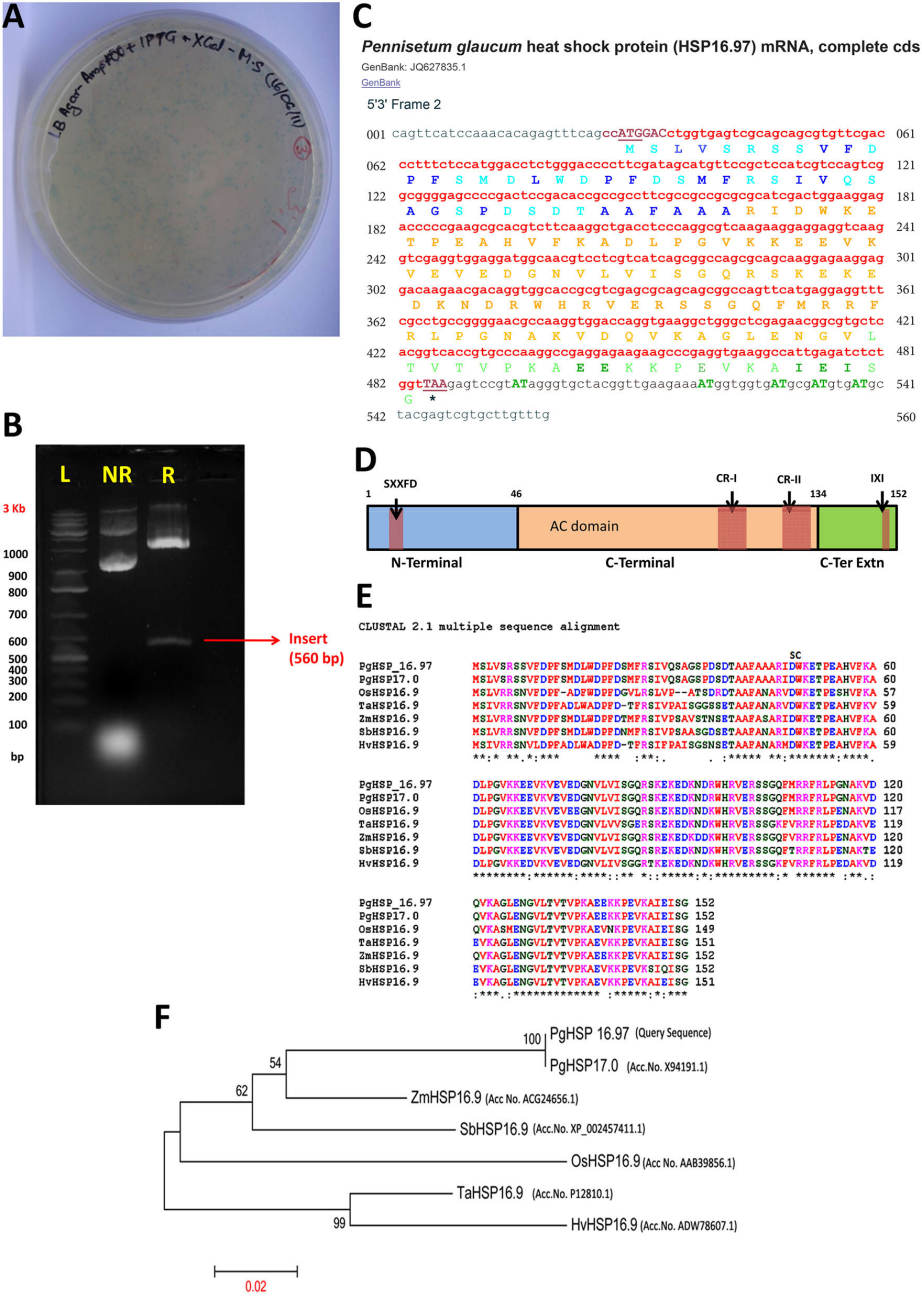
Cloning, validation, and structural analysis of *PgHSP16.97*. **(A)** Blue-white screening of positive cloning of insert. The *PgHSP16.97* gene was eluted from agarose gel and cloned onto pGEM®-T easy cloning vector. The transformed cell culture was plated onto LB agar plates containing ampicillin, IPTG, and X-Gal and incubated overnight at 37°C. White colonies represent positive cloning. **(B)** Restriction enzyme digestion of the recombinant pGEM®-T easy plasmid with *PgHSP16.97* gene *M* = 1 kb ladder; lane 1 (NR) = non-restricted plasmid; lane 2 (R) = EcoR-I restricted plasmid confirming the presence of insert fragment of size 560 bp. **(C)** The complementary DNA (cDNA) nucleotide sequences, wherein the coding region (letters in red bold font), 5′, 3′ untranslated region (UTR) regions (letters in black regular fonts). Translation start site and termination codon are underlined, polyadenylation signals (repeated “AT”-rich DNA nucleotide sequences. Various functional domains in the sequence have been significantly marked, such as variable N-terminal domain (light blue font) with hydrophobic groups represented by dark blue font, alpha crystalline domain (ACD; yellow font), and c terminal extension (green font) in which glutamic acid residues and “I X I” residues are represented (in bold green font). **(D)** Schematic representation of *PgHSP16.97* protein structure, including three motifs: the N-Terminal (Blue box), ACD (orange box), and the c-terminal extension (green box), with SXXFD motifs, Conserved regions (CR-I and CR-II) and IXI motifs. **(E)** Alignment of class-I cereal HSP17 amino acid sequences-*PgHsp16.97* (this study), *PgHSP17* (X94191.1), *OsHSP 16.9* (AAB39856.1), *TaHSP16.9* (P12810.1), *ZmHsp16.9* (ACG24656.1), *SbHSP16.9* (XP_002457411.1), and *HvHSP16.9* (ADW78607.1) in which SC= start of carboxyl-terminal domain in each alignment, which is the most conserved region of the alpha-crystallin/small heat shock protein (HSP) family; * = conserved residue, : = conserved residue with strongly similar property, = conserved residue of weakly similar property (ClustalW; www.ebi.ac.uk). **(F)** Phylogenetic tree constructed using the ClustalW software showing *PgHSP16.97* and its inferred evolutionary relationships with HSP 17 genes identified from different crops/cereals (https://figshare.com/s/ef60263530940ec529f4).

Full-length HSP20 ORF of 560 base pairs (bp) and 152 amino acids with an apparent molecular weight of 16.97 kDa and an estimated isoelectric point of 5.79 was isolated and cloned from the pearl millet heat-tolerant genotype WGI-126, named as *PgHSP16*.97, and deposited with the accession number JQ627835.1 to the NCBI nucleotide database. The homology search that was done by using the deduced amino acid sequence of *PgHSP16.97* against the translated non-redundant nucleotide database suggested that the *PgHSP16.97* was related to the other eukaryotic sHSPs and showed an overall 100–88% sequence identity with sHSPs of *Cenchrus americanus* (Acc no. CAA63901.1), *Z. mays* (Acc no. *NP_001150783.1*), *Setaria italica* (Acc no. XP_004968025.1), *Saccharum* hybrid cv. *ROC22* (Acc no. AFK73383.1). The presence of ACD found in alpha-crystalline-type sHSPs and a similar domain found in p23 (a co-chaperone for HSP90) and in other p23-like proteins confirmed that the isolated sequence belongs to the sHSP gene family (Figure 3).

The ProtComp (http://linux1.softberry.com/cgi-bin/programs/proloc/protcomppl.pl) analysis produced an integral subcellular localization prediction score of 9.9 for cytoplasmic location, which indicated that *PgHSP16*.97 belongs to class I sHSP. It also carried a nuclear localization sequence. The *PgHSP 16.97* (Figure 3D) monomer contains a variable N-terminal domain (aa, 1–46), the conserved HSP20 or ACD (aa, 47–134), and a less variable C-terminal extension (aa, 135–152). The organelle-localized sHSPs have the necessary transit, targeting, or signal located on N-terminal of protein, which was absent in the sequence-indicating cytoplasmic localization. There is considerable diversity in HSP 17.0 evolution in cereals, with a few conserved motifs and regions, such as IXI motifs, consensus region I (CR-I) (P-X_14_-GVL), CR-II (P-X_15_-V-L), R residue at position 114 in C-terminal, and SXXFD motif at N-terminal, which were the signature regions of cytosolic sHSP. Only a few highly conserved domains at N-terminus (21/46) were observed during sequence alignment among cereal class I sHSP. The *PgHSP 16.97* shows close similarity to *PgHSP 17.0 (X94191.1)* and is evolutionarily very close to *Zea HSP 16.9* proteins and is more divergent to that of sorghum.

### Expression Pattern of PgHSP16.97

**Figure 4:**
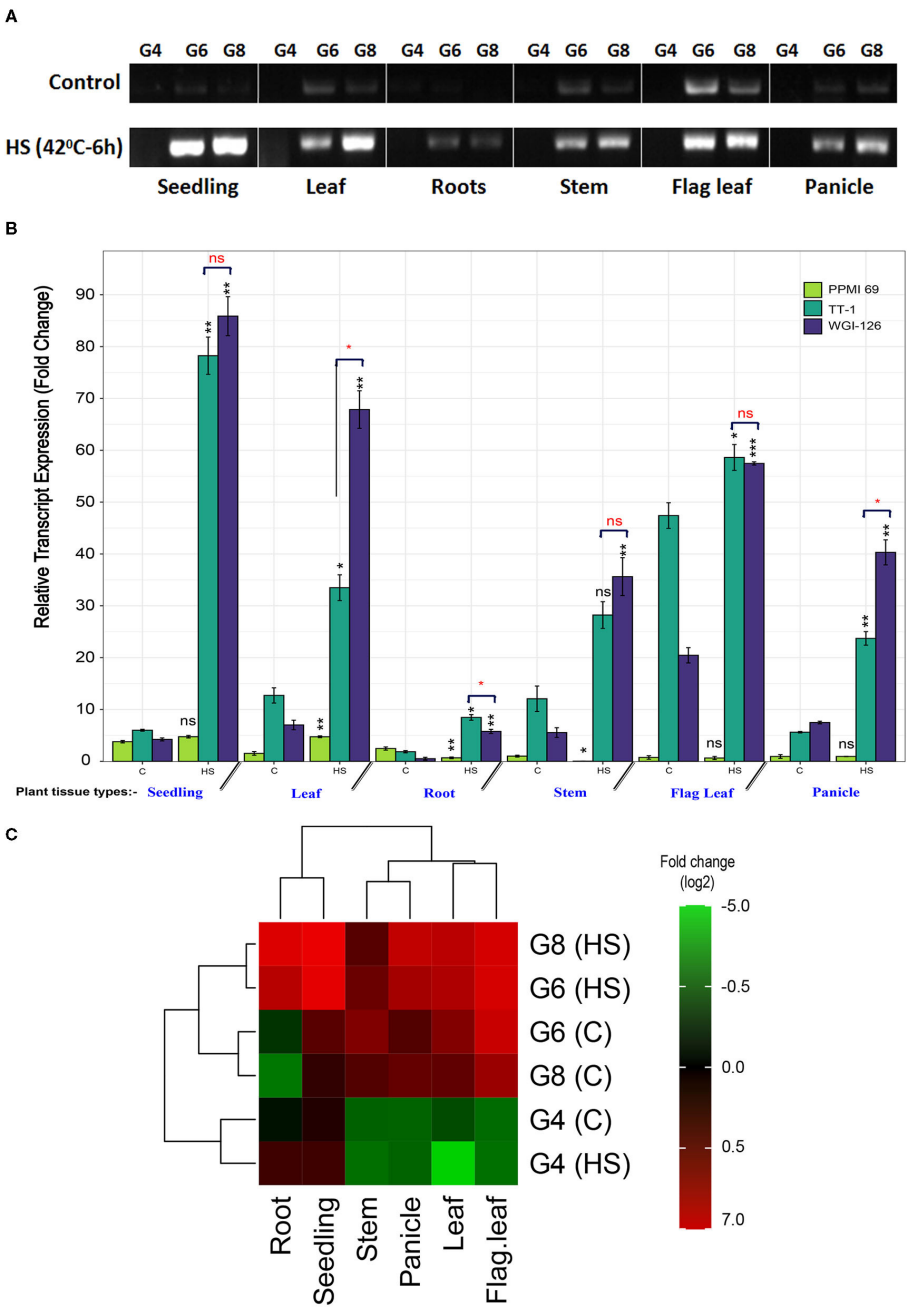
**(A)** Semiquantitative RT-PCR profile of *PgHSP16.97* transcripts after heat stress (HS) (42°C for 6 h) on different plant tissues of the plant in three contrasting genotypes. The presented results are representative of at least three independent biological replicates. [PPMI-69 (G4), TT-1 (G6), and WGI-126 (G8)]. Relative transcript expression profiling of *PgHSP16.97* for heat tolerance (mean ± SE) among three contrasting genotypes obtained by quantitative real-time PCR (qRT-PCR). For relative quantification, the least expression value from a control in the sensitive cultivar was taken as unity and the relative fold changes in expression were calculated for the remaining genotypes and the treatments. The paired *t*-test was done to compare the genotypic means between the control and heat-stressed sample to determine the significance of upregulation/downregulation of gene (indicated in black font). Also, the difference in mean among the tolerant genotypes was done to determine a significant difference in the transcript accumulation among the thermotolerant genotypes (red fonts). Significance codes: ***p ≤ 0.001, **p ≤ 0.01, *p ≤ 0.05, ns, not significant. **(C)** Heat map of *PgHSP16.97* has been generated based on the log2-transformed fold-change values in the heat-stressed sample (HS) when compared with its control sample **(C)**. The column of the heat map represents the plant tissue from where the samples were taken, and rows represent a combination of genotypes and treatments. The relative values for each point are depicted by color intensity, with green indicating the downregulation and red indicating the upregulation of *PgHSP16.97* gene. The color scale for log2-transformed fold-change values is shown in the right side (https://figshare.com/s/4fbc26be5c90b74f521d).

RNA from six different tissues of the contrasting genotypes, *viz.*, PPMI-69 (thermosusceptible), TT-1, and WGI-126 (thermo-tolerant genotypes), were collected to analyze and understand the expression pattern of the cloned gene *PgHSP16.97* under different developmental stages. Figure 4A shows the intensity of PCR products as measured by densitometry. Upon heat stress, the relative expression level of *PgHSP16.97* increased significantly (*p* < 0.05) in different tissues of genotypes except in seedlings, flag leaf, and panicle of susceptible genotype PPMI-69. It had the least expression of PgHSP16.97 in different tissues at different stages during qRT-PCR analysis (Figures 4B,C). Among the thermotolerant genotypes, WGI-126 had the highest expression at the seedling stage and was 19 times higher than the expression in the corresponding control. In addition, WGI-126 showed a significant difference in the expression concerning another thermotolerant genotype TT-1, in the case of the transcript expression at leaf, root, and panicle under heat stress. In general, the transcript expression of the *PgHSP16.97* was higher in seedlings (10.5 times), followed by roots (4.4 times), leaves (4.1 times), and least in flag leaves (0.6 times) (Table 6).

**Table 6:** Comparison of the relative gene expression level of PgHSP16.97 at different tissues among the contrasting pearl millet genotypes under heat stress treatment.

**Samples**	**Genotype**	**Fold change**	**Gene expression**	**Tolerant vs. Suceptible**		**B/w Tolerant**	
				**t-stat**	***p*-value**	**t-stat**	***p*-value**
Seedling	PPMI 69	0.26	Upregulation	−3.84	0.062	−1.16	0.366
	TT-1	12.05	Upregulation	−21.15	0.002		
	WGI-126	19.31	Upregulation	−20.33	0.002		
Leaf	PPMI 69	2.11	Upregulation	−12.02	0.007	−5.86	0.028
	TT-1	1.64	Upregulation	−6.96	0.020		
	WGI-126	8.67	Upregulation	−16.24	0.004		
Root	PPMI 69	−0.73	Down regulation	11.32	0.008	5.11	0.036
	TT-1	3.57	Upregulation	−9.13	0.012		
	WGI-126	10.37	Upregulation	−9.73	0.010		
Stem	PPMI 69	−0.96	Down regulation	5.10	0.036	−1.80	0.214
	TT-1	1.34	Upregulation	−3.21	0.085		
	WGI-126	5.43	Upregulation	−11.03	0.008		
Flag leaf	PPMI 69	−0.13	Down regulation	0.16	0.889	0.53	0.652
	TT-1	0.24	Upregulation	−4.14	0.050		
	WGI-126	1.81	Upregulation	−25.92	0.001		
Panicle	PPMI 69	0.00	Constant	0.00	0.999	−4.76	0.041
	TT-1	3.23	Upregulation	−12.49	0.006		
	WGI-126	4.39	Upregulation	−14.05	0.005		

### Homology Modeling of *PgHSP16.97*

**Figure 5:**
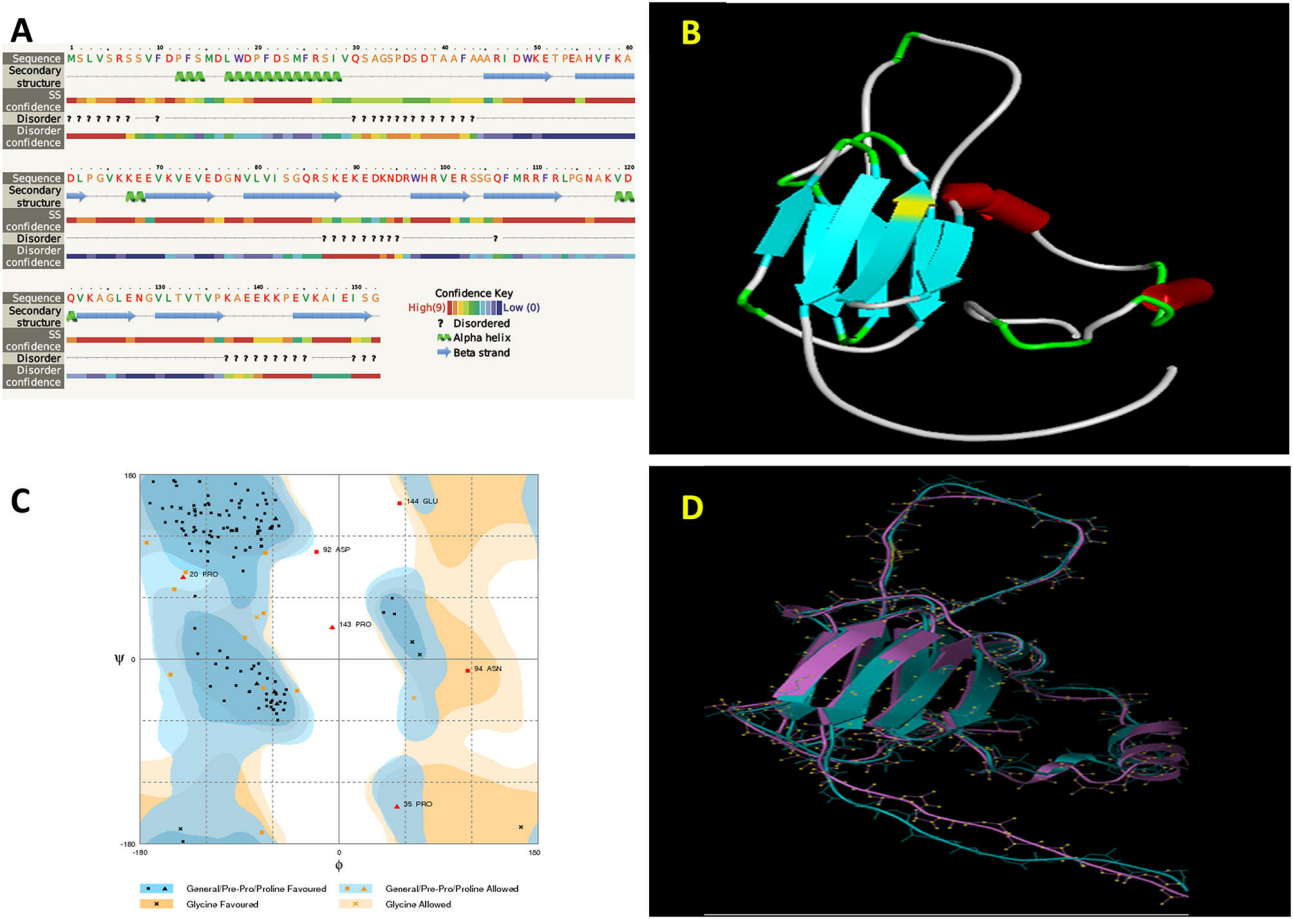
Geometrical and three-dimensional (3D) structural analysis of *PgHSP16.97* protein **(A)**. Secondary structure analysis of *PgHSP16.97* has been shown by using the Phyre2. **(B)** Predicted 3D structure of PgHSP16.97 given by the modeler 9v11 showing the arrangements of alpha and beta chains. **(C)** Ramachandran plot of *PgHSP16.97* generated by Rampage showing dihedral (phi and psi) angles in which favored region marked as dark blue and orange and allowed regions marked as light blue and orange shades whereas the residue on white spaces are the outliers. **(D)** Structural superimposition between model and template protein (1GME) given by STRAP (Blue: Template and Violet: model) (https://figshare.com/s/13b4394e6e6137f826ce).

The functionality of a protein largely depends on the structural features such as a motif, domain, and their 3D confirmations. The deduction of its 3D structure from its primary structure has been considered as one of the major goals to understand the structural dynamics of the protein. Proteins are known to be dynamic entities in cellular solutions. Homology modeling is one of the approaches that are followed for the 3D structure prediction of proteins based on their match with the primary amino sequence of the experimentally solved homologous protein. In our studies, *PgHSP16.97* shared 80% similarity in their primary amino acid sequence levels with the wheat *HSP16.9* protein (PDB no: 1GME). Hence, the crystal structure of the wheat *HSP16.9* protein (PDB no: 1GME) was chosen as a template for building a *PgHSP16.97* model using the program Modeller 9v11 (Sali and Blundell, 1993). The secondary structure of the protein consists of four alpha-helices and nine beta-strands (Figure 5A). The Accelrys Discovery Studio 3.5 Client program was used to depict the *PgHSP16.97* molecular model (Figure 5B). Five models of 3D structures of *PgHsp16.97* were generated at various refinement levels and validated by using the program Procheck. The best model with a Procheck score of −0.09 was selected (Figure 5C). Superimposition of the model with the template and root mean square deviation (RMSD) calculation was carried out using the program (http://www.bioinformatics.org/strap/). The RMSD value of the selected PgHSP 16.97 model structure is 1.60 A° regarding the template 1GME. The structural superimposition was done with the STRAP interface and was observed to have a better level of the superimposition of the model onto the template, as shown in Figure 5D.

### Chromosomal Distribution and Structural Analysis of *PgHSP20* Genes

**Figure 6:**
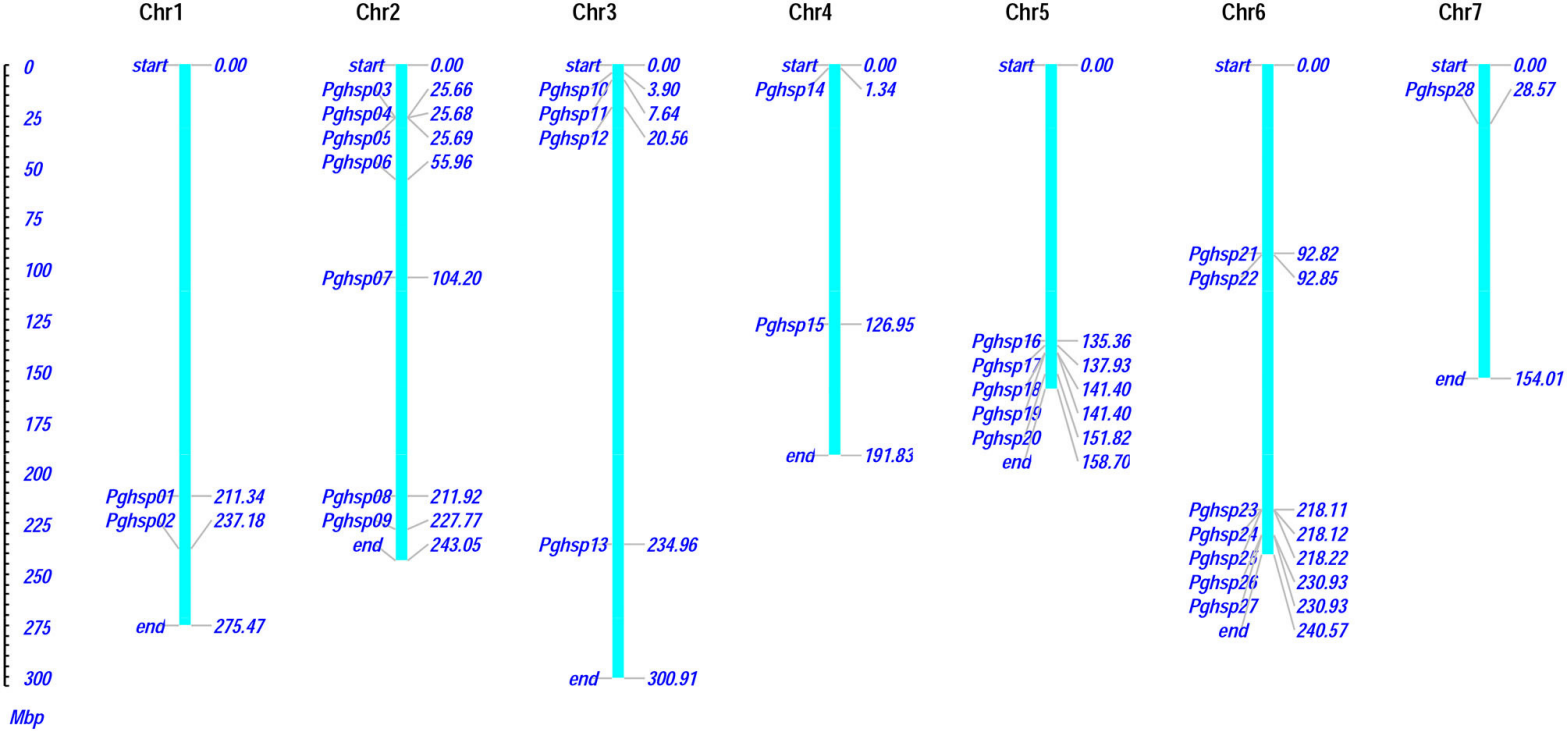
The chromosomal distribution and positioning of *PgHSP20s* across all seven chromosomes of pearl millet. Seven chromosomes with varying lengths are shown in the million base pair (Mbp) scale in the left, where the chromosome (represented in bars) is marked by the respective genes with its genomic position (https://figshare.com/s/d113cd59ea02544c8825).

**Figure 7:**
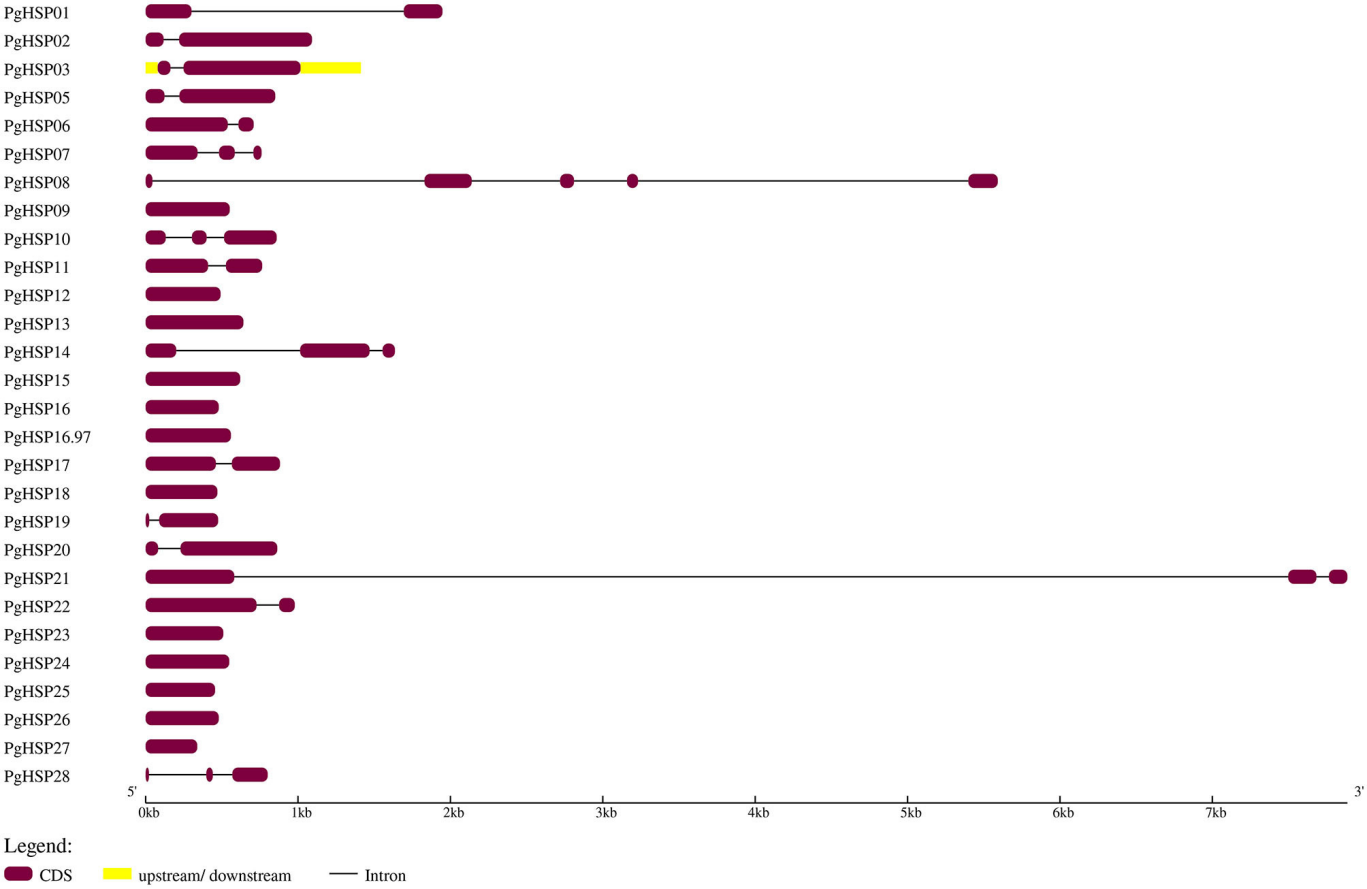
Structural elucidation of the identified 28 *PgHSP20* genes. The diagram represents the exon and intron present in genomic DNA, in which exons were represented by violet boxes (https://figshare.com/s/504eb0133d9e684232e7).

The present investigation revealed that *PgHSP 16.97* is located on chromosome 6 and their ortholog in the pearl millet genome revealed that 28 genes were evenly distributed across the genome (Supplementary Table 2). A variation in gene length of HSP20 varies from 338 bp (*PgHSP03*) to 17.6 kbp (*PgHSP27*). The chromosomal location with its coordinates is shown in Figure 6. Chromosomes 2 and 6 have most of the HSP20 genes (7), most of which are located on the telomeric regions, whereas chromosome 7 has the least one. The structural features of the identified genes were elucidated by using the Gene Structure Display Server (GSDS). Figure 7 shows a varying pattern of total exonic and intronic regions among the 28 *PgHSP20* genes. Among 28 genes, 11 genes (39.28%) possess only a single exon, 10 genes (35.71%) have two exons and a single intron, 14.28% of the genes have three exons and two introns, and the remaining one gene, and *PgHSP02* has multiple exons of five.

### Phylogenetic Analysis of *PgHSP20* Gene Family

**Figure 8:**
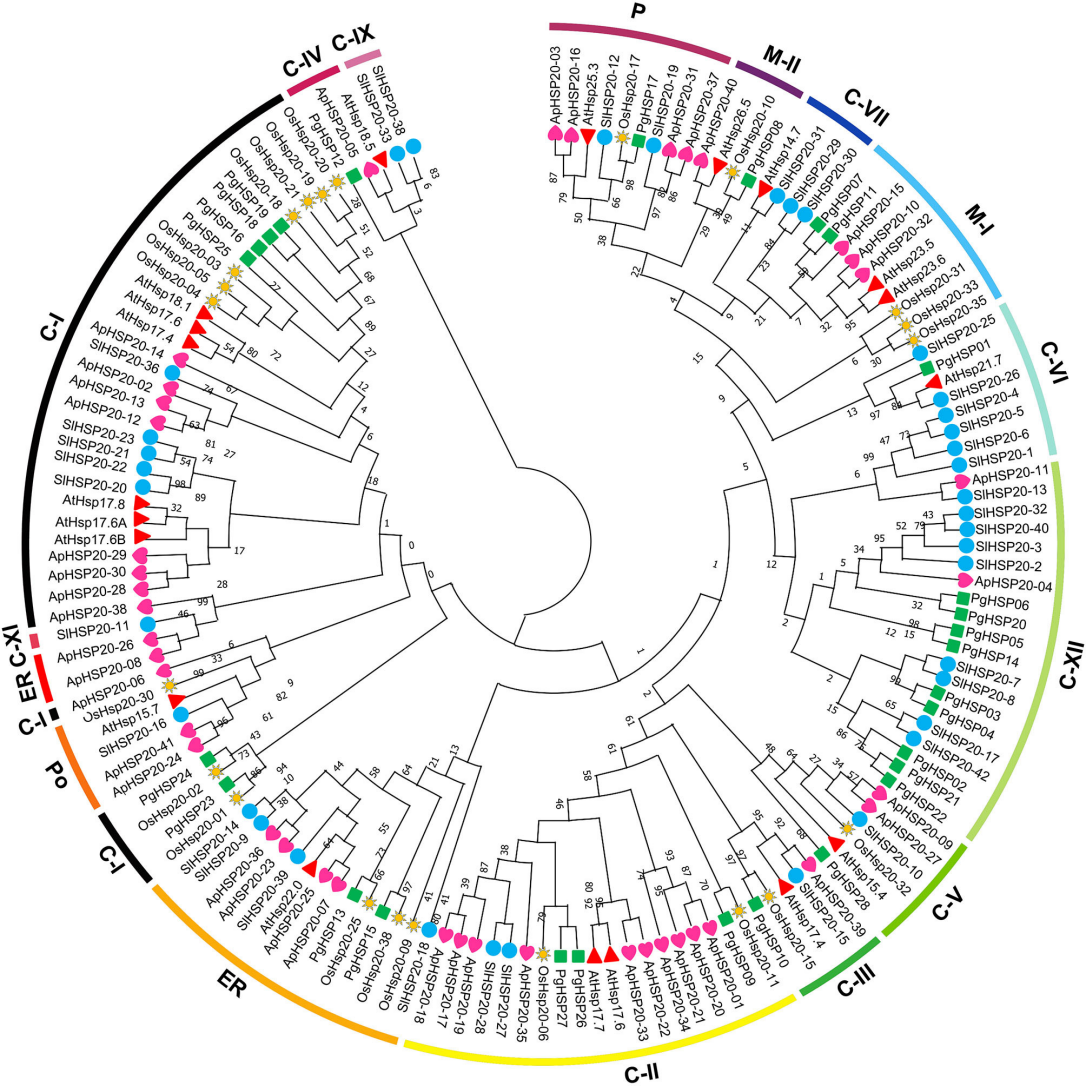
Phylogenetic analysis of representative *HSP20* proteins from *Arabidopsis*, rice, tomato, and apple along with the 28 predicted HSP20 genes from the pearl millet genome. The phylogenies were constructed by using MEGA.X by the maximum likelihood (MLE) method with a 1,000-bootstrap replication. The 16 subfamilies were distinguished with different colored arcs (https://figshare.com/s/17c47e8fe8fa000e992f).

A phylogenetic tree was constructed based on the amino acid sequences of HSP20 genes (Figure 8), 28 from pearl millet (*P. glaucum*), 22 from rice (*O. sativa*), 19 from *Arabidopsis thaliana*, 37 from tomato (*Solanum lycopersicon*), and 41 from apple (Supplementary Table 3). A total of 147 HSP20s were divided into 16 subfamilies, 35 cytosol Is (C-Is), 19 C-IIs, 5 C-IIIs, 3 C-IVs, 6 C-Vs, 8 C-VI, 4 C-VII, 10 mitochondria Is (M-Is), 4 M-IIs, 5 peroxisomes (Po), 15 from the endoplasmic reticulum (ER), and 9 from plastids (P) based on the phylogeny and the subcellular localization. The new classes were also divided as follows: 2 proteins in C-IX, 1 in C-XI, and 20 in C-XII. However, the PgHSP20 proteins were distributed into 12 out of 16 subfamilies, in which nine proteins were in C-XII, six in C-I, three in C-II, two each in M-I and ER, one each in C-III, C-IV, C-V, C-VI, M-II, and P based on the phylogeny and the subcellular localization. Out of 28 HSP20s, 22 belonged to the cytosol, which suggested that the cytosol might be the functional site of the plant HSP20s.

### Digital Expression Profile of *PgHSP20s* in Response to Heat Stress

**Figure 9:**
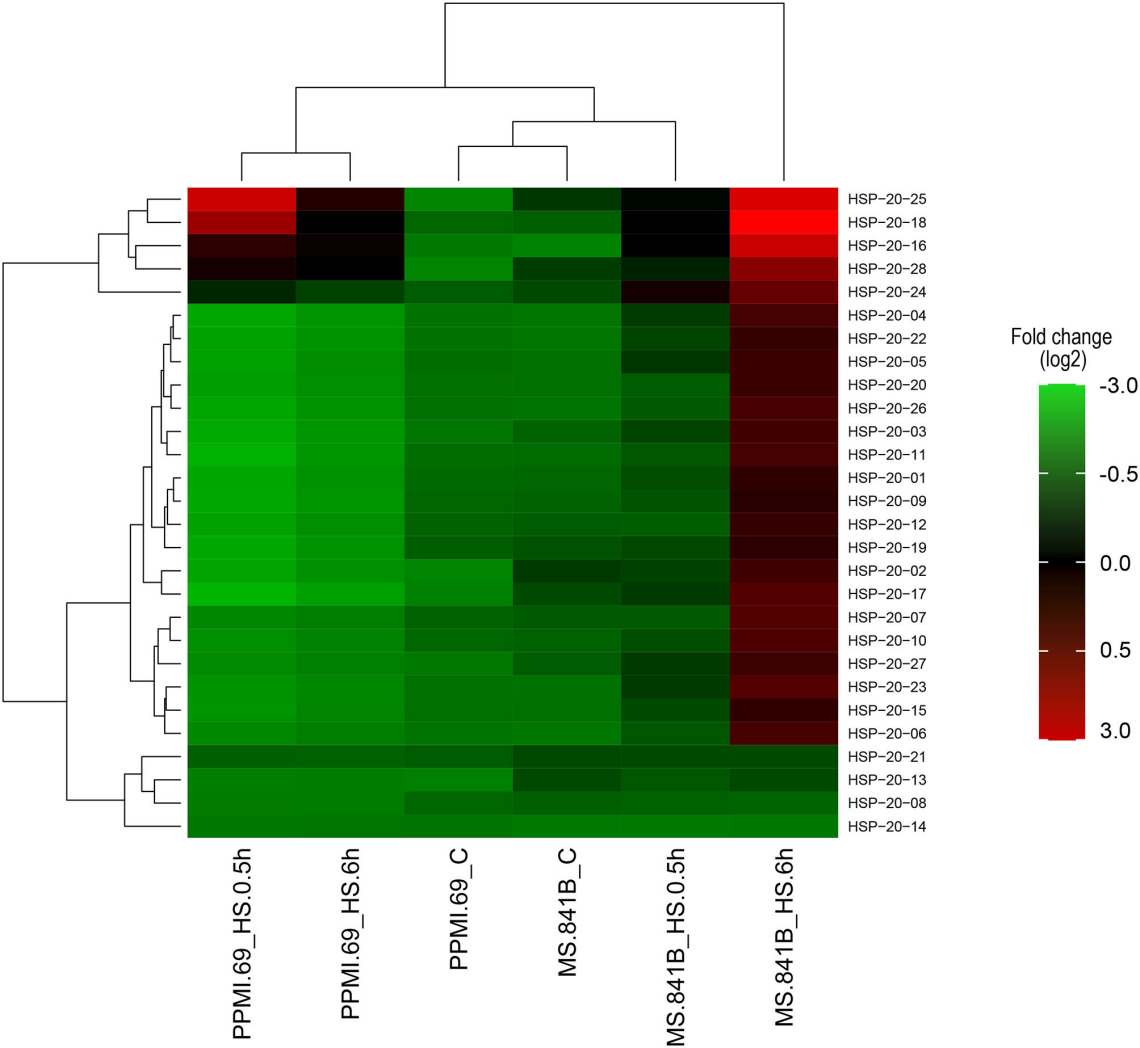
The expression heat map of 28 pearl millet *HSP20* genes under heat stresses based on the RNA-sequencing (RNA-Seq) data. The heat map with clustering was created by using a log2-based Fragments Per Kilobase of transcript per Million mapped reads (FPKM) value. On the right side of the heat map with a color scale representing relative expression values. C, control plants were maintained at 25°C; HS, heat stress of 42°C for 0.5 and 6 h, respectively, was imposed on plants maintained in the growth chamber (https://figshare.com/s/7132de92e289274bc8e1).

To further explore the expression profiles of *PgHSP20* genes in response to heat stress, whole-genome RNA-Seq libraries, including three independent biological replicates of two contrasting genotypes with the control and heat-treated set, were downloaded from NCBI (SRP151237) and analyzed. The Fragments Per Kilobase of transcript per Million mapped reads (FPKM) values from the RNA-Seq data were used to estimate the expression levels of 28 pearl millet *HSP20* genes and were further employed in the creation of expression heat map (Figure 9). The expression analysis of the contrasting genotypes of pearl millet under the various degrees of heat stresses has shown a highly uneven expression pattern by most of the *PgHSP20* genes. The heat map also showed that the 28 *HSP20* genes clustered in three groups. Cluster A contains five members (HSP20-16, 18, 24, 25, and 28), which were highly expressed upon heat stress compared with the control in both tolerant and susceptible genotypes. All 19 members from cluster B were mainly upregulated after 6 h of heat stress in stress-tolerant genotypes alone. The remaining four members (8, 13, 14, and 21) grouped in cluster C were barely upregulated upon heat stress as compared to the control in both contrasting genotypes.

## Discussion

Heat shock proteins are a collection of molecular chaperones that have been shown to have a critical role in plant stress reaction as well as growth and development of plants. Small HSPs are the largest and diverse group of HSPs among the plants that possess definite roles in various abiotic stress responses (Scharf et al., 2001; Ouyang et al., 2009; Sarkar et al., 2009; Yu et al., 2016; Zhao et al., 2018; He et al., 2019; Ji et al., 2019; Yao et al., 2020), but they are poorly studied in case of pearl millet.

### Response of Pearl Millet Genotypes to Heat Stress

The study was initiated with an analysis of some selected physio-biochemical parameters among the eight pearl millet genotypes. Significant differences were observed among the genotypes, treatments, and their interaction for all the studied physio-biochemical parameters. Upon heat stress, the value of MSI, RWC, and SPAD was diminished among the genotypes and prominent in thermosusceptible genotypes, such as PPMI-69, whereas the level of lipid peroxidation increased among the genotypes upon heat stress due to the formation of free radicals and ROS. Thermo-tolerant genotypes, such as WGI-126 and TT-1, showed limited changes to the abovementioned parameters in response to heat stress and maintaining cellular homeostasis. High genetic variability among the pearl millet genotypes using physiological parameters, such as MSI, RWC, and TBARS, for seedling thermotolerance was observed in earlier reports by Mukesh Sankar et al. (2014); James et al. (2015), and Maibam et al. (2020).

Heat stress leads to the induction of various thermotolerant genes, such as HSFs and HSPs, in pearl millet genotypes. Earlier research experience on the responses of HSP70 and HSF in pearl millet seedlings to heat stress in the gene expression level (Maheswar Rao et al., 2010; Reddy et al., 2010; Divya et al., 2019; Sun et al., 2020) suggested that heat stress response could be used as a molecular marker to identify heat-responsive genotypes. In the current experiment, the expression pattern suggested the upregulation of the transcript level of heat-responsive genes, such as *Pgcp70* and *PgHSF*, under high-temperature stress, supporting the earlier findings of the heat-induced HSPs (Howarth. C. J., 1991; Mishra et al., 2007; Sun et al., 2020). It was also noticed that, under a normal growth condition (25°C), there was a slight accumulation of these transcripts as HSP 70 and its master regulator, HSFs, have a critical role in seed germination and development and also could be attributed to the inherent thermotolerance in pearl millet cultivars as shown by the result of the MSI (Maibam et al., 2020). The *Pgcp70* was expressed in small quantity under normal conditions but was enhanced with heat treatment. A large quantity of HSP 70 in the early stage of heat exposure (2 h) indicates a major role in heat stress during the early phases of heat stress. Later on, the transcript level decreases as stress is continued (6 h), which indicates that an immediate shock in the genotype leads to a higher and rapid induction of Pgcp70, the level of which diminishes with continuous exposure to heat stress, suggesting constant involvement of HSP in heat shock for a longer duration (Waters et al., 1996). It was also noticed that the Pgcp70 transcript accumulation showed a slight increase from 7- to 10-day-old seedlings, which indicated that plants tend to increase the temperature tolerance with their development. The expression of the *PgHSF* was at much lower levels when compared to the transcript level of *Pgcp70*. Even such a low-level HSF transcript could trigger the transcription of *Pgcp70* under heat stress. The transcript expression of the HSF gene in pearl millet showed an utmost rise in the transcript level within 30 min of exposure and gradually came down with time upon heat stress. This indicated its stress regulatory or initiator role. This result was in agreement with the study of Reddy et al. (2010). The expression profile of *PgHSF* was nearly constant for a long period of heat exposure for 2 h and 6 h in comparison with the expression profile of *Pgcp70*. *PgHSF* transcript abundance under high-temperature treatment in the seedling stage was less than *Pgcp70* with the same condition, which suggests an initiator role towards rapid stress response in germinating seedling.

The expression of *Pgcp70* and *PgHSF* was found to be associated with the changes in MSI and TBARS under heat stress. Both parameters had shown a low-to-moderate significant correlation with the gene expression of both marker genes. Some studies suggested that the HSP co-inducer hydroximic acid derivatives intercalate into the membrane and stabilize their lipid rafts by regulating membrane structure and composition (Balogi et al., 2019). Similarly, Usman et al. (2018) reported the role of HSP70 in membrane stability in chili pepper. Also, the accumulation of ROS upon heat stress leads to the accumulation of thiobarbituric acid derivatives leading to membrane lipid damages, and subsequently the electrolyte leakage (Killi et al., 2020). The extent of differences observed in HSP70 expression, membrane stability, and lipid peroxidation can be used to improve the thermotolerance capacity of the genotypes. The differential expression pattern was observed for these two heat-responsive genes among different genotypes. WGI-126 showed a positive response to heat stress by accumulating more transcripts whereas PPMI-69 exhibited a very low accumulation of transcripts, as indicated by the physio-biochemical parameters. These results suggested that transcript expression profiling can be used for screening thermo-tolerant and susceptible lines in a large pool of genotypes along with the identification of the potential genes responsible for thermotolerance at various stages of crop growth.

### Isolation and Characterization of *PgHSP16.97* Under Heat Stress

Among the molecular chaperones, the sHSPs were diverse and were found in both prokaryotes and eukaryotes, which were usually untraceable under a normal physiological situation in plant cells, but they were induced upon stress, leading to plant tolerance to stress, such as drought, poor plant nutrient availability, salinity, and extreme temperatures (Singh et al., 2016). Hence, the diversification and abundance of sHSPs in a plant are thought to reflect the adaptation of a plant to heat stress (Pareek et al., 2021). Among the HSPs, HSP20 forms the first line of defense against stress in the cell during heat stress. They have the ability to bind to the denatured or partially folded proteins to avoid irreversible unfolding or erroneous protein aggregation or to bind to unfolded proteins in an energy-free manner until suitable conditions for renewed cell activity arise, allowing further refolding by HSP70/HSP100 complexes, earning the nickname “paramedics of the cell” (Hilton et al., 2013). Hence, we have isolated and cloned full-length cDNA encoding for HSP 17.0 from *P. glaucum* to understand the structural signature present in this protein for its role in heat tolerance among genotypes. The analysis of nucleotide and deduced amino acid sequence of the cDNA clone revealed the presence of alpha-crystalline-type sHSPs. A similar domain found in p23 (a co-chaperone for Hsp90) and other p23-like proteins confirmed that the isolated sequence belongs to the sHSP gene family. Alpha-crystalline occurs as large aggregates, comprising two types of related subunits (A and B) that are highly similar to the small HSPs (11–35 kDa), particularly in their C-terminal halves. Alpha-crystalline has chaperone-like properties, such as the ability to protect proteins from precipitation, thereby improving the cellular tolerance to stress. The model structure revealed that the N-terminal arm of the *PgHSP16.97* represents an extensive, intrinsically unstructured domain rich in hydrophobic residues (53%), which will play key roles in protein–protein interactions with denatured proteins and thus critical to substrate interactions (Jaya et al., 2009). Due to its ability to present multiple binding site conformations, PgHSP16.97 is highly effective at interacting efficiently to protect a wide range of critical cellular proteins. In addition, N-terminal regions are important for stabilizing an oligomer through interlocking subunits by forming two disks intertwined to form pairs of knot-like structures, and the hydrophobic contacts in these knots are buried inside an oligomer (van Montfort et al., 2001). The C-terminal extension is variable in length, and its function in those cellular compartments is enigmatic. As per the sequence information, the C-terminal extension was rich in glutamic acid residues (E-), which were critical for its chaperone activity (Morris et al., 2008). Moreover, there was an Ile-X-Ile residue at the C-terminal extension (β_10_ strand), which has a role in the oligomerization of HSPs (Studer et al., 2002) by interacting with the hydrophobic pockets formed at β_4_ and β_8_ of ACD strands. Two CRs within C-terminal separated by a region with hydrophilic residues form a signature sequence for the identification of cytosolic plant sHSP. The CR-I consists of residues (P-X_14_-GVL) that are involved in the multimerization of *PgHSP17* subunits, and the CR-II consists of residues (P-X_15_-V-L) involved in the solubility of the protein complex. Arginine (R) conserved across the cereal sHSP gene at position 114 is responsible for the stabilization of dimer by the formation of an intermolecular salt bridge with glutamic acid at position 100 (van Montfort et al., 2001).

Moreover, the gene expression pattern in pearl millet suggested its constant involvement in response to heat stress at different developmental stages. Under controlled conditions, the expression level of PgHSP16.97 was very low, still showing a higher expression in flag leaf, stem, and developing panicles, which indicated the involvement of HSP20 genes in plant reproductive tissue development. Cytosolic class I HSP20s will begin to accumulate on flag leaf and develop seed from mid-maturation throughout the late maturation under normal conditions. A similar observation concerning the HSP20 genes was reported by Poidevin et al. (in press) while conducting riboprofiling studies on developing pollens and in developing wheat panicles (Wang et al., 2020). Upon heat stress, the expression of the gene was upregulated by up to 19 times in almost all tissues of tolerant genotypes. However, the expression was found to be higher in the seedling stage as compared to other tissues. Young seedlings are prone to dehydration by heat stress than other tissues due to the presence of more meristematic tissues. Several studies have speculated that cytosolic class I HSP20s may function to protect cellular components during seedling desiccation (Kalemba et al., 2019).

### Genome-Wide Scan and Expression Profiling of Pearl Millet HSP20 Gene Family in Response to Heat Stress

In the current investigation, we used the pearl millet genome database to identify 28 HSP20 genes and investigated their sequence characteristics. The number of HSP20 genes in pearl millet was higher than that in *Arabidopsis* (19) (Scharf et al., 2001), but slightly lower than that in tomato (37) (Yu et al., 2016), foxtail millet (37) (Singh et al., 2016), and rice (39) (Ouyang et al., 2009), and much lower than that in sorghum (47) (Nagaraju et al., 2020) and bread wheat (163) (Muthusamy et al., 2017). The gene duplications and rearrangements that occurred during evolution are quicker to attribute for this difference (Devos et al., 2000; Varshney et al., 2017). The expansion of the number of gene families in plants is thought to be aided by gene duplication and the same with the evolution of sHSPs (Waters and Vierling, 2020). The pearl millet HSP20 family could be divided into 12 subfamilies according to the phylogenetic analysis, which is consistent with the previous evolutionary classification of HSP20 genes in sorghum and foxtail millet (Singh et al., 2016; Nagaraju et al., 2020). A total of 22 out of 28 members belong to nucleo-cytoplasmic subfamilies, and among these subfamilies, CXII was the largest subfamily with 20 members. As a result, we hypothesized that the cytoplasm, as a site primarily for protein synthesis, could be the primary location for HSP20 interacting with the denatured proteins to prevent them from aggregating and degrading inappropriately (Waters and Vierling, 2020). The gene structure analysis indicated that most pearl millet HSP20 genes (74.99%) have no introns or a single intron, suggesting relatively simple gene structures. The gene structure analysis indicated that nearly 75% of the pearl millet HSP20 genes were either intronless or with a single intron, implying relatively simple gene structures. This is concurrent with the results of the previous finding of the researchers, which showed that the plants were prone to retain more genes with no intron or a short single intron (Ji et al., 2019). According to the RNA-Seq atlas, temporal regulation of the PgHSP20 gene family was observed under heat stress among the contrasting genotypes. The heat map showed that the *PgHSP20* genes clustered in three groups, in which cluster A contains five members (HSP20-16, 18, 24, 25, and 28), which were highly expressed upon heat stress compared with the control in both tolerable and susceptible genotypes. These genes have no introns except for *PgHSP-28*. Furthermore, we also found that these PgHSP20 genes were more heat stress-inducible in susceptible genotypes compared to tolerable genotypes, as evidenced by their expression pattern after 30 min of heat stress induction. The rapid response of sHSPs upon heat stress in susceptible genotypes over tolerant genotypes for a short period of stress was also observed in crops (Chakraborty et al., 2018).

## Conclusion

HSP20 genes are a large and diverse group of genes involved in plant stress responses, and as a result, they have been studied extensively in a variety of crop plants. However, to the best of our knowledge, there are a handful of reports available in otherwise naturally stress-tolerant model C_4_ crops such as pearl millet. An attempt was made in the current study to validate the powerfulness of the expression pattern study as a molecular screening technique in identifying the contrasting genotypes based on the expression of heat-responsive marker genes, such as HSP or HSF, in the seedling stage of pearl millet. Using the transcript expression-based screening, one gene (PgHSP16.97) belonging to the HSP20 family was identified, cloned, and characterized for the first time from pearl millet cv. WGI 126. Later, this sequence information was used in the identification of the genome-wide distribution of HSP20 genes. Isolating and identifying these functional genes will help researchers in gaining an in-depth understanding of the molecular genetic basis of pearl millet stress adaptation and genetic improvement. It can serve as powerful genomic resources for genetic engineering. Also, the *in silico* structure prediction can provide further insights into the molecular regulation of stress tolerance, thereby bridging them together to fight against the unpredicted nature of abiotic stress.

## Data Availability Statement

The datasets presented in this study can be found in online repositories. The names of the repository/repositories and accession number(s) can be found in the article/Supplementary Material.

## Author Contributions

SM performed the investigation and wrote the manuscript. CT and SB were involved in the conceptualization, supervision, and editing of the manuscript. SB helped in validation, funding acquisition, and editing of the manuscript. SPS and CB were involved in the editing of manuscript. SLS performed the data analysis and edited the manuscript for final submission. All authors contributed to the article and approved the submitted version.

## Conflict of Interest

The authors declare that the research was conducted in the absence of any commercial or financial relationships that could be construed as a potential conflict of interest.
