# Supplementary figures and images for: Differential Modulation of Heat-Inducible Genes Across Diverse Genotypes and Molecular Cloning of a sHSP From Pearl Millet [Pennisetum glaucum (L.) R. Br.]
Source: Front Plant Sci. 2021 Jul 16;12:659893. doi: 10.3389/fpls.2021.659893 (PMC8324246; doi:10.3389/fpls.2021.659893)

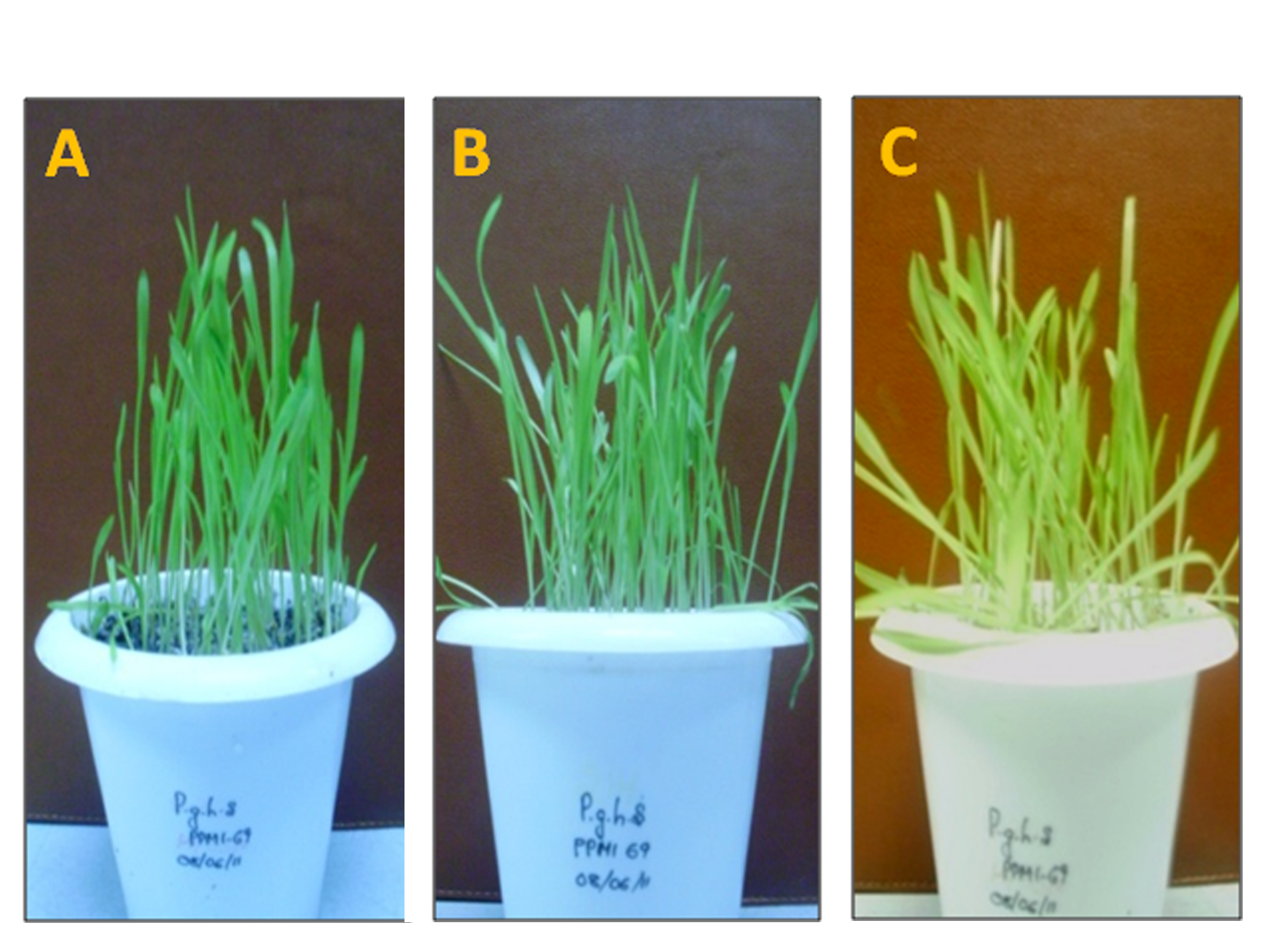

Supplement: Supplementary file 4 [file Image_1.TIF]

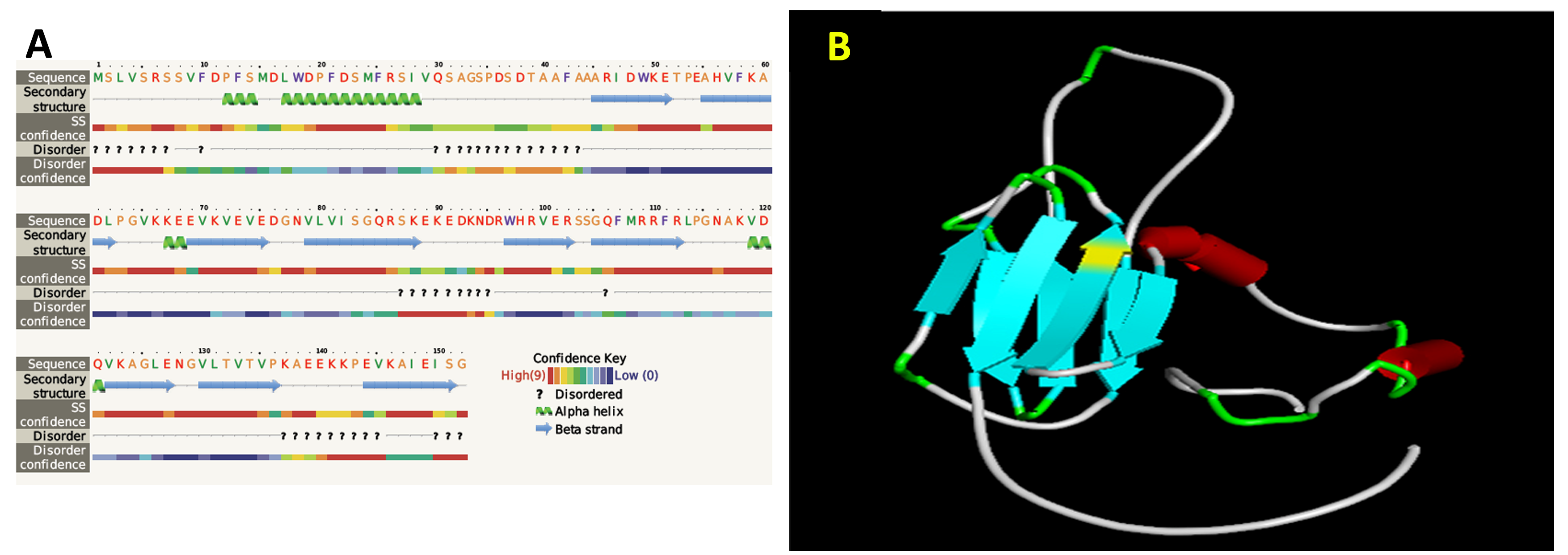

Supplement: Supplementary file 5 [file Image_2.TIF]
